# Antioxidant and* Ex Vivo* Immune System Regulatory Properties of* Boswellia serrata* Extracts

**DOI:** 10.1155/2017/7468064

**Published:** 2017-03-13

**Authors:** Daniela Beghelli, Gloria Isani, Paola Roncada, Giulia Andreani, Onelia Bistoni, Martina Bertocchi, Giulio Lupidi, Alessia Alunno

**Affiliations:** ^1^School of Biosciences and Veterinary Medicine, University of Camerino, Camerino, Italy; ^2^Department of Veterinary Medical Sciences, University of Bologna, Ozzano Emilia, Italy; ^3^Rheumatology Unit, Department of Clinical and Experimental Medicine, University of Perugia, Perugia, Italy; ^4^School of Pharmacy and Health Product Sciences, University of Camerino, Camerino, Italy

## Abstract

*Boswellia serrata* (BS) is an important traditional medicinal plant that currently represents an interesting topic for pharmaceutical research since it possesses several pharmacological properties (e.g., anti-inflammatory, antimicrobial, and antitumour). The safety and versatility of this dietary supplement should allow for its use in numerous pathological conditions; however the quality of the extracts needs to be standardized to increase the clinical success rate resulting from its use. In the present study, different commercially available* B. serrata* extracts were employed to compare their AKBA content and in vitro antioxidant power. Furthermore, their ability to modulate the immune system regulatory properties was investigated. Our results showed that the AKBA content varied from 3.83 ± 0.10 to 0.03 ± 0.004%, with one sample in which it was not detectable. The highest antioxidant power and phenolic content were shown by the same extract, which also exhibited the highest AKBA concentration. Finally, the BS extracts showed the ability to influence the regulatory and effector T-cell compartments. Our results suggest that frankincense should be further investigated for its promising potentiality to modulate not only inflammation/oxidative stress but also immune dysregulation, but attention should be paid to the composition of the commercial extracts.

## 1. Introduction

The gum resin of* Boswellia serrata* (BS), a traditional treatment of Ayurvedic medicine in India also identified as Indian frankincense, Salai Guggal, or Indian olibanum, has been used for centuries as a remedy for many health problems [1].

Indeed, the anti-inflammatory, antiarthrogenic, and analgesic activities of its dried resinous gum (guggulu), derived from tapping the* Boswellia* tree, have been recognized since ancient times [2]. The inflammatory response represents the first-line defense of the body to tissue damage and/or to microbial invasion and it determines the recruitment of immune cells and some plasma proteins [3]. The final goal of inflammation is healing, elimination of the external or internal inflammation* noxae,* and the restoration of homeostasis.

This immune response should be self-limiting but the persistence of the stimulus in predisposed subjects leads to chronification of the process and eventually to irreversible tissue injury. Indeed, persisting low-grade inflammation plays a key role in the pathogenesis of many chronic diseases [4] and most of these diseases are also associated with increased production of reactive oxygen species (ROS), which results in oxidative stress [5]. Therefore, inflammation is tightly linked with oxidative stress [6] by an interdependent relationship and both participate in the pathogenesis of many chronic diseases [4].

During recent decades, many authors have investigated the mechanisms of action of BS extracts related to the inflammatory process. Studies in animal models showed that the ingestion of a defatted alcoholic extract of* Boswellia* decreased polymorphonuclear leukocyte infiltration and migration as well as primary antibody synthesis [7, 8] and led to almost total inhibition of the classical complement pathway [9]. In vitro studies revealed that the boswellic acids, a group of pentacyclic triterpenoid compounds, and their acetylated derivatives inhibit the biosynthesis of leukotrienes, the proinflammatory 5-lipoxygenase products which cause increased permeability [10], in a dose dependent manner [11]. In addition, Cuaz-Pérolin et al. [12] observed that 3-acetyl-11-keto-beta-boswellic acid (AKBA) was a natural inhibitor of the transcription factor NFkB, whose presence is a prerequisite for the formation/action of cytokines/chemokines involved in inflammatory reactions.

Therefore, these natural compounds can dampen the inflammatory response, but also simultaneously reduce oxidative stress, as observed by Umar et al. [13].

In recent years, extracts from the gum resin of BS have been shown to target both the humoral and adaptive immune responses [14] eventually interfering with the inflammatory cascade [15].

However, to the best of our knowledge, no studies have yet investigated whether the BS extracts can exert any effects on specific T-cell subsets whose balance is crucial for the maintenance of immune homeostasis: the regulatory T-cells (Tregs) and the proinflammatory Th1/Th17 cells. In the present study, different commercially available* B. serrata* extracts were employed as follows: (i) to compare their composition and in vitro antioxidant power; (ii) to test their ability to modulate Treg/Th1/Th17 cells* ex vivo*.

## 2. Materials and Methods

### 2.1. Chemicals and Plant Material

All chemicals used were of analytical reagent grade from Sigma-Aldrich (St. Louis, MO, USA). Six (A, B, C, D, E, and F, resp.) of the seven BS oleogum resins utilized in the present study were commercially available and were certified for a content of boswellic acids of 65%. The pale yellow or white amorphous powders were insoluble in water but soluble in methanol and dimethyl sulfoxide (DMSO). The seventh (G) BS extract was an aqueous extract obtained by a process of bioliquefaction based on enzyme biocatalysis [16] and was kindly offered by its producer (Phenbiox, Calderara di Reno, Bologna, Italy).

### 2.2. TLC Analysis

BS extract separation was performed on 20 × 20 cm silica gel plates with a fluorescent indicator at 254 nm (Sigma-Aldrich, St. Louis, MO, USA). Pentane and diethyl ether (2 : 1) containing 1% (v/v) of acetic acid were used as a mobile phase. Twenty mg of BS extracts was dissolved in 300 *μ*L of ethanol, sonicated for 5 minutes, and centrifuged. Clear supernatant (10 *μ*L) was carefully layered at 1.5 cm from the bottom of the plate giving an elution distance of 9 cm. After the separation, plates were observed at 254 nm and developed with anisaldehyde (5 mL) in glacial acetic acid (50 mL) and H_2_SO_4_ (1 mL). The TLC analysis of BS G extract was not performed due to its particular formulation.

### 2.3. HPLC-DAD Analysis

A HPLC system (Beckman Coulter, Brea, CA, USA), comprising a 116 pump, a 507 automatic autosampler, an UV-Diode Array 168 detector, and integration software 32 Karat, was used for the analysis of seven BS extracts. Samples were prepared by dissolving extracts in methanol. Briefly, the separation was performed using a reverse phase column Luna C18 5 *μ*m 250 × 4.6 mm (Phenomenex, Torrance, CA, USA) with a guard column PR C-18 5 *μ*m 15 × 4.6 mm (Phenomenex, Torrance, CA, USA). Chromatographic separation was achieved in isocratic conditions at room temperature. The mobile phase was a mixture of phosphoric acid (H_3_PO_4_ 10 mM in water) and acetonitrile (19 : 81 v/v). The flow rate was 1 mL/min, and the injection volume was 50 *μ*L. The analyses were made at two different wavelengths (210 and 260 nm) and UV spectra were recorded in the range of 190–300 nm. A standard stock solution was prepared by dissolving 5 mg of AKBA analytical standard (Sigma-Aldrich, 5 mg, batch number BCBN2928V, CAS number 416619) in methanol (5 mL). The calibration curve was obtained by analyzing nine serial dilutions (50 ppm, 25 ppm, 15 ppm, 10 ppm, 7.5 ppm, 5 ppm, 2.5 ppm, 1 ppm, and 0.5 ppm) of the stock solution and by plotting the peak area measured at 260 nm against AKBA concentrations. The following equation of the curve was obtained:

(1) $$\begin{array}{c} y=79739x-5414,\;R^{2}=0.999. \end{array}$$

The AKBA peaks were identified on the basis of the retention time on the chromatogram at 260 nm. All measurements were performed in triplicate and data were reported as mean ± SD.

### 2.4. Quantification of Total Phenolic Content (Folin-Ciocalteu Method)

The total content of polyphenolics was determined by a colorimetric method as described by Singleton and Rossi [17] and adapted to a 96-well plate format. Briefly, the seven BS extracts were redissolved in 1 mL of methanol and 100 *μ*L/well of each extract was dispensed into a flat bottom 96-well tissue culture plate (Becton Dickinson, Lincoln Park, NJ); then 150 *μ*L Folin-Ciocalteu reagent (1 mL Folin-Denis' reagent in 4 mL H_2_O) was added. The plate was incubated for 10 min at 37°C. Next, 50 *μ*L of a saturated Na_2_CO_3_ solution in H_2_O was added to each well and the plate was incubated for a further 10 min. The absorbance was measured at 765 nm. A standard calibration curve was plotted using gallic acid (0–300 mg/L). The results were expressed as g of gallic acid equivalents (GAE) per g of dry weight of BS extract. The results were expressed as the average of three measurements.

### 2.5. Antioxidant Activity

Free radical scavenging activity was studied using 1,1-diphenyl-2-picrylhydrazyl (DPPH) on a microplate analytical assay according to the procedures described by Srinivasan et al. [18], while the total radical scavenging capacity of the same products was measured by the 2,2-azinobis-(3-ethylbenzothiazoline-6-sulfonic acid) (ABTS) assay as modified by Re et al. [19], for application to a 96-well microplate assay.

Finally, the determination of antioxidant activity by FRAP assay was carried out according to the procedure described by Müller et al. [20], monitoring the reduction of Fe3^+^-tripyridyl triazine (TPTZ) to blue-colored Fe2^+^-TPTZ.

Trolox was used as standard in all assays and the ability of BS extracts to scavenge the different radicals was expressed as tocopherol-equivalent antioxidant capacity (mmol TE/g of product) and, for DPPH and ABTS assays, also as IC_50_, the latter defined as the concentration of the tested material required to cause a 50% decrease in initial DPPH/ABTS concentration. All measurements were performed in triplicate and reported as mean ± standard deviation (SD).

### 2.6. Immune Responses

#### 2.6.1. Cell Proliferation Assay

Human peripheral blood mononuclear cells (PBMCs) of seven healthy donors (HD; 5 male and 2 female, age mean ± standard deviation: 47 ± 12.1 years) were isolated from fresh heparinized venous blood (10 mL/HD) by gradient separation. The study was approved by the local ethics committee (CEAS Umbria) and written informed consent was obtained from participants in accordance with the Declaration of Helsinki. The final concentration of live cells was adjusted to 1 × 10^6^/mL in complete medium (RPMI-1640 medium containing 10% heat-inactivated serum, L-glutamine (2 mM), Euroclone®, penicillin (100 U/mL), and streptomycin (100 *μ*g/mL), Biochrom^AG^, Berlin). PBMCs were stained with carboxyfluorescein diacetate succinimidyl ester (CFSE) cell tracer (BioLegend, San Diego, CA), dispensed into flat bottom 24-well tissue culture plates (Becton Dickinson, Lincoln Park, NJ) (1 mL/well), and cultured for 5 days at 37°C in 5% CO_2_. For proliferation stimuli were either 1 *μ*g/mL of pokeweed mitogen (PWM; Sigma-Aldrich Co. Ltd., Saint Louis, Missouri) or 1.2 *μ*g/mL of phytohemagglutinin (PHA; Biochrom^AG^, Berlin), in the presence or absence of two BS extracts (0.1 *μ*g/mL). BS extract A revealed the highest in vitro antioxidant power; BS extract G was obtained with a different extraction method compared to other BS compounds (by Phenbiox srl). So lymphocytes were exposed, in the culture medium, to an AKBA concentration of 3.8 ng/mL that resulted below the mean maximal concentration of 6 ng/mL detected in human plasma after an oral administration of a BS dry extract in fasted condition [21].

A negative control was represented by PBMC cultured without any mitogen/extract (C), so that the base proliferation could be estimated [22]. Therefore, nine different experimental theses for each blood sample were tested. Flow cytometry analyses were performed on a standard FACSCalibur™ flow cytometer (Becton Dickinson, Mountain View, CA) running the CellQuestPro™ software. The results of the lymphocyte proliferation assay were expressed as a percentage (%). Furthermore, the lymphocyte proliferation index (LPI) was calculated with the following formula:

(2) $$\begin{array}{c} \text{LPI}=\frac{\left(\text{FP}-\text{BV}\right)}{\text{BV}}*100, \end{array}$$

where the FP values are represented by the “final percentages” of cell proliferation (after 5 days in culture with BSs and with/without the mitogens), whereas the BV values are represented by the “basal values” obtained by cells either stimulated (with PHA and PWM) or not (CTR) with the mitogen but without BSs [23].

#### 2.6.2. Phenotypic Characterization of Peripheral Blood Mononuclear Cells

PBMCs obtained from the seven volunteers were seeded further (1 × 10^6^ cells/well) into additional flat bottom 24-well tissue culture plates and cultured, with or without PHA, for 5 days at 37°C in 5% CO_2_. After culture, six-hour in vitro stimulation with 25 ng/mL phorbol 12-myristate 13-acetate (PMA), 1 *μ*g/mL ionomycin, and 1 *μ*L/mL BD GolgiPlug™ (BD Biosciences) in complete medium was performed. For surface staining, fluorescein isothiocyanate (FITC), Pe-Cy7, or APC labelled antihuman CD4, CD3, and CD25 and respective isotypes were used (BD Biosciences, San Jose, CA, USA, Immunotools). Then, cells were permeabilised with 0.1% saponin blocking buffer after 4% paraformaldehyde fixation to perform intracellular staining with Alexa Fluor 647 or Phycoerythrin (PE) antihuman IL-17 and INF*γ*, and their isotype controls were used (BD Biosciences). When required, cells were permeabilised with commercially available Forkhead box protein P3 stain buffer (BD Biosciences) for intracellular staining with PE-labelled mAb to human FoxP3 and respective isotype controls [24]. Debris was excluded by backgating to CD3 T-cells in forward scatter/side scatter (FSC/SSC) plots. Samples were analyzed using FACSCalibur flow cytometer (BD) and CellQuestPro software (BD).

### 2.7. Statistical Analysis

The results of immune responses are reported as mean ± standard error of the mean (SEM) from seven samples of different HD. The unpaired Student's *t*-test was used to compare biological data from controls with that from BS A or BS G treated samples, respectively (GraphPad Prism, 2007) [25]. *p* values < 0.05 for two-tailed test were considered statistically significant.

## 3. Results

### 3.1. TLC Chromatograms

**Figure 1:**
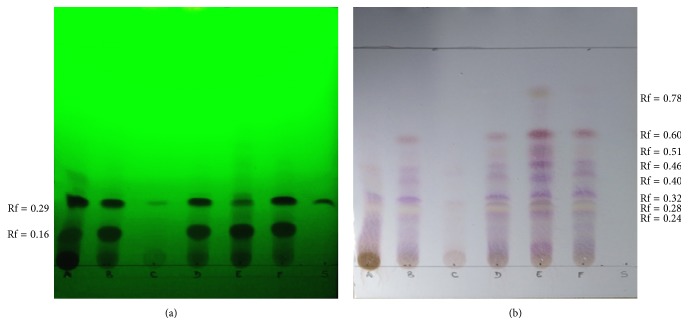
UV detection at 254 nm (a) for UV-active boswellic acids. Chromatograms after dyeing with anisaldehyde (b). Rf values are reported for the most relevant spots. A–F: six different powder extracts of* Boswellia serrata* gum resin; S: 3-acetyl-11-keto-beta-boswellic-acid (AKBA) analytical standard (Rf = 0.29).

The preliminary qualitative TLC screening of the BS extracts is shown in Figure 1. The UV analysis revealed two main spots visible in all samples, with the exception of extract C. The first spot had a Rf of 0.16, whereas the second spot had a Rf of 0.29. The use of the AKBA standard (lane S) allowed for the identification of this boswellic acid in the spot with Rf of 0.29. It is noticeable that extract C lacked the first spot and presented only traces of the spot corresponding to AKBA, while extract A presented the greatest spot referring to AKBA. Extracts A and E presented other components absorbing at 254 nm.  Figure 1(b) demonstrates the pattern obtained after dyeing with anisaldehyde, heating, and color development. Similar profiles were obtained for extracts B, D, E, and F. Instead, extract E was characterized by a major number of spots; extract A lost the spots at the higher Rf; and extract C was found to have fewer components. The majority of samples shared the spots detected at Rfs of 0.24, 0.32, 0.40, 0.46, and 0.60.

### 3.2. HPLC-DAD Analysis

At 260 nm, the majority of extracts presented two major peaks: the first one, at Rt of 13.2 min, and the second one, identified as AKBA by the use of an analytical standard, at Rt of 26 min. Other minor peaks were also present. AKBA concentrations for each sample, calculated on the basis of the peak area and the calibration curve, are shown in Table 1. Extract A presented significantly higher amounts of AKBA as compared with the other samples. Extract C presented only a small peak of AKBA and was lacking the first peak.

**Figure 2:**
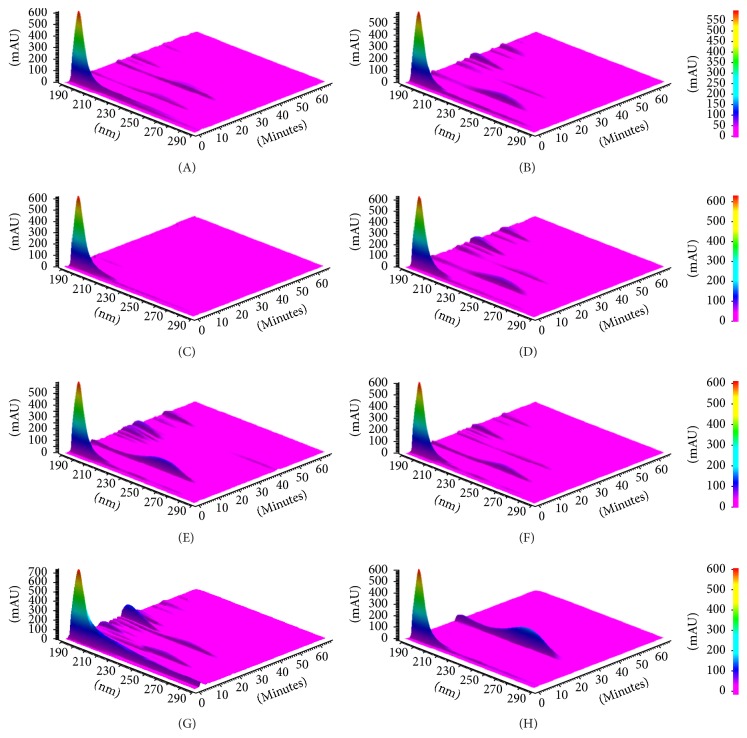
Chromatograms of* Boswellia serrata* extracts (BS) after HPLC-DAD analysis. BS (A)–(F) extracts were diluted 1 : 400 in methanol, whereas, BS (G) extract was diluted 1 : 20 in methanol. The chromatogram of the AKBA analytical standard is also reported (H). The absorbance (mAU) is reported on the *y*-axis, wavelength (nm) on *x*-axis, and the retention time (minutes) on the *z*-axis.

Other components in the BS extracts were visualized at 210 nm. The chromatograms of all the BS extracts analyzed at the different wavelengths to highlight the variability of the components present in the samples are reported in Figure 2.

### 3.3. Determination of Total Phenolic Content and Antioxidant Capacity of* Boswellia serrata* Extracts

All the BS extracts utilized in the present study exhibited a relatively low content in phenolics ranging from 7.68 ± 0.9 mg gallic acid equivalent (GAE)/g (extract A) to 0.11 ± 0.05 mg GAE/g (aqueous extract G) (Table 2). The chemical complexity of the extracts, often mixtures of many compounds with differences in functional groups, polarity, and chemical behavior, could lead to scattered results, depending on the antioxidant test employed. For these reasons, in the present study, the BS extracts were screened for their free radical scavenging and reducing properties through three test systems: (a) 1,1-diphenyl-2-picrylhydrazyl (DPPH) radical scavenging, (b) monitoring of the reduction power of Fe3^+^ (FRAP assay), and (c) evaluating the total radical scavenging capacity (ABTS assay). All the BS extracts analyzed showed relative radical scavenging activities in all the assays employed, revealing antioxidant powers lower (from 34 to nearly 580 times) than that of Trolox (positive control, Table 2).

However, between the BS extracts investigated, the BS extract A showed the highest scavenging reducing power and the highest polyphenolic content.

### 3.4. Immunomodulatory Activity

**Figure 3:**
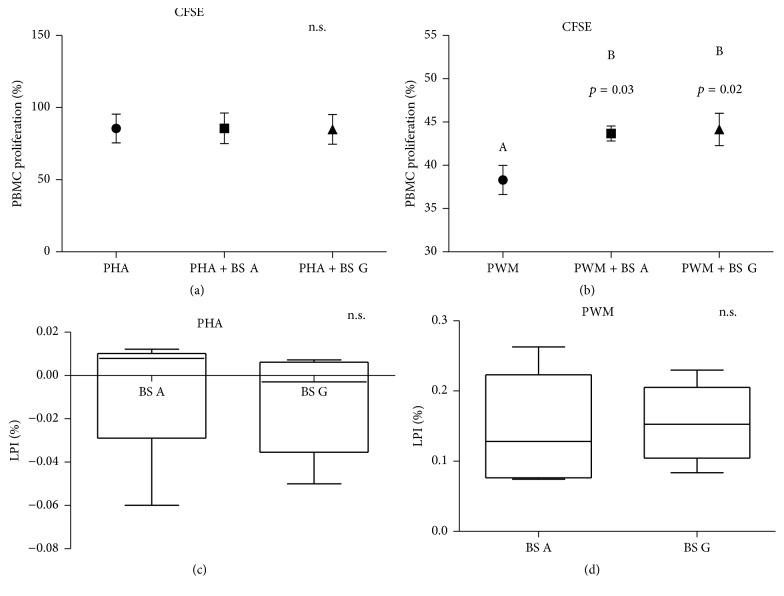
*Boswellia serrata* (BS) extract (A or G) effects on lymphocyte proliferation assay. Data are shown as mean ± SEM of seven independent experiments. PBMCs were cultured with phytohemagglutinin (PHA; graphics (a) and (c)) or pokeweed mitogens (PWM; graphics (b) and (d)) and stained with carboxyfluorescein diacetate succinimidyl ester cell tracer (CFSE). The lymphocyte proliferation index (LPI) was calculated as reported in the text. ^A,B^Different letters for *p* < 0.05. n.s. = not significant.

The in vitro lymphocyte proliferation (CFSE assay) was not influenced by the BS extracts if cells were cultured without any activator (data not shown) or if stimulated by PHA. However, when cells were activated by PWM, the addition of BS extracts induced a significantly higher lymphocyte response (Figures 3(a) and 3(b)). No significant differences were observed between the two types of BSs for the LPI (Figures 3(c) and 3(d)).

**Figure 4:**
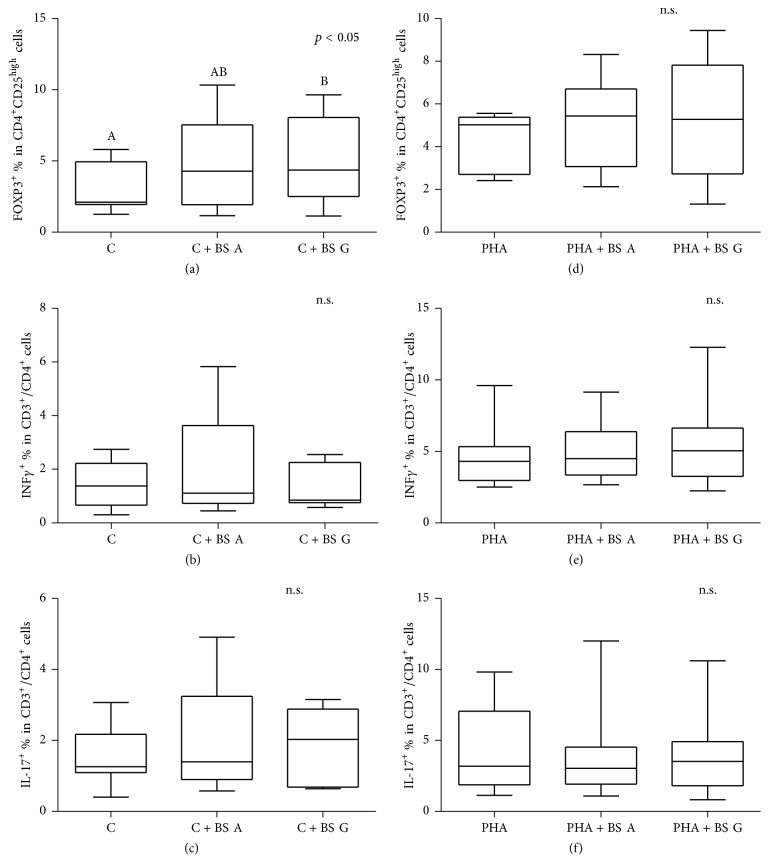
*Boswellia serrata* (BS) extract (A or G) effects on Treg (CD4^+^CD25^+^FOXP3^+^ cells), Th1 lymphocyte (INF*γ*^+^ cells), and Th17 cell (IL-17^+^) responses. Data are shown as mean ± SEM of seven independent experiments. PBMCs were cultured in absence (controls, C; graphics (a), (b), and (c)) or presence of mitogen (phytohemagglutinin, PHA; graphics (d), (e), and (f)) and with/without BS extracts. ^A,B^Different letters for *p* < 0.05. n.s. = not significant.

The in vitro regulatory or Th1/Th17 proinflammatory responses (Figures 4(d), 4(e), and 4(f)) were not significantly modulated by the addition of the BS extracts when PBMCs were triggered by PHA. Neither type of utilized extract (A versus G) elicited an altered response.

However, when cells were not PHA pulsed, an increase of FOXP3^+^ cells was observed in PBMCs cultured with the BS extracts. In particular, a tendency towards a higher number of regulatory cells was observed for extract A (*p* = 0.079), whereas extract G led to a significant increase of FOXP3^+^ cells (*p* = 0.045; Figure 4(a)).

Furthermore, higher number of Th17^+^ cells, although not significant, was again observed when PBMCs were cultured with the extract G (Figure 2(C)).

## 4. Discussion

The therapeutical efficacy of BS extracts has been extensively investigated in arthritis, asthma, diabetes mellitus, colitis, and cancer [26, 27] in light of their antioxidant and anti-inflammatory activities [28]. Indeed, all these diseases share a persistent dysregulation of redox status that contributes to the intensity and duration of the inflammatory response and therefore to the induction and perpetuation of chronic inflammation.

It is noteworthy that the phytochemical content of* B. serrata* oleogum resin is dependent on both the botanical origin and the geographical origin [29]. Usually it consists of 30–60% triterpenes (such as *α*- and *β*-boswellic acids, lupeolic acid), 5–10% essential oils, and 20–35% polysaccharides [30]. According to Singh et al. [31], in aqueous and ethanolic extracts of* B. serrata*, it is possible to recognize alkaloids, carbohydrates, phytosterols, terpenoids, phenolic compounds, flavonoids, and tannins. However, other authors found also glycosides, proteins, and saponins [32].

Furthermore, the wide variations of pharmacologically active molecules in commercial BS formulations could significantly affect the final product [33].

The first aim of this study was to perform a comparative analysis on the composition of different dry and aqueous extracts of* B. serrata* gum resin as a tool for the evaluation of the quality of the extracts.

Combination of TLC and HPLC analyses can be considered as a multidimensional analytical approach combining fast qualitative screening with an accurate and precise quantification of specific compounds. We decided to quantify AKBA because the boswellic acid, characteristic and unique to* Boswellia* genus, is considered the most effective, at least in* in vitro* studies. When analyzed at 260 nm, four of the seven samples presented comparable profiles, characterized by the presence of two main peaks the second of which corresponds to AKBA. The other components of BS extracts, lacking the keto moiety, were visualized only at a less specific wavelength (210 nm) as already reported [34]. The AKBA concentrations detected in samples A, B, D, and F are similar to those reported by other authors [30, 35], while those found in extracts C and G were 10 or 100 times lower, respectively. It can be hypothesized that extract C belongs to* Boswellia* species other than* B. serrata*, due to a wrong botanical identification by local producers. Indeed, it has already been reported that the elution profile of* Boswellia frereana* gum resin lacks KBA and AKBA peaks [34], while* Boswellia sacra* gum resin contains much lower amounts of KBA and less AKBA than* B. serrata* [34, 36]. Concerning extract G, this aqueous extract showed a lower content of AKBA, but it was enriched by other components of the phytocomplex, as demonstrated by the additional peaks obtained in the elution profile at 210 nm. The presence of these compounds is probably related to the particular and innovative extraction method [16].

The composition of BS extract E is challenging, due to the discrepant results in TLC and HPLC analysis. The spot at Rf of 0.26, a putative AKBA component, was not confirmed by a corresponding peak in the HPLC chromatogram at 260 nm. Other components of the phytocomplexes containing a keto moiety should have contributed to this spot and further analyses are needed to identify these molecules.

The antioxidative potential and radical scavenging activity of aqueous and ethanolic extracts of* B. serrata* are significantly correlated to their total phenolic and flavonoid content [31].

According to Kohoude et al. [37], the amount of phenolics in* Boswellia* genus (315 g/kg) is comparable to reference extracts rich in phenolic compounds. Despite what is reported in literature, the BS samples investigated here were all characterized by either relatively low antioxidant properties or total phenolic content. Indeed, the latter ranged from 0.11 ± 0.05 to 7.68 ± 0.9 mg GAE/g versus values of 28.46–12.73 mg GAE/g obtained by other authors in aqueous and ethanolic extracts, respectively [31].

According to the extraction procedure, the antioxidant activity increased with the polarity of the solvent. Other authors reported that the essential oil of* Boswellia dalzielii* was characterized by low antioxidant activity [36] and that this was due to the extraction method adopted (e.g., low polarity of the solvent) that determined the absence of phenolics, especially flavonoids.

In the present study, the extraction methods adopted by manufacturers or even the preservative systems used could be responsible for the low level of total phenolic and flavonoids compounds, which are contained mainly in the volatile essential oil component of the oleogum resin.

Furthermore, although theoretically the aqueous extract (extract G) should have characteristics closest to the natural product being obtained by an enzymatic hydrolysis that maintains the intact phytocomplexes [16], we observed that it was the one with the lowest antioxidant properties and total phenolic contents.

However, these results are at least partially in line with those of other authors reporting that the wild habitat samples, with a completely different profile as compared to the market samples, were those lacking antioxidant activity [28].

Nevertheless, the BS extracts of the present study were able to significantly modulate some immune responses investigated independently of the in vitro antioxidant activities. As reported in Figure 3(b), when cells were stimulated by PWM, a mitogen that stimulates B lymphocytes in the presence of T-cells, the PBMC proliferation was significantly increased (*p* < 0.05) by the addition of the BS extracts (0.1 *μ*g/mL for both extracts A and G) and the LPI did not change between the two BS extracts (Figure 3(d)).

Conversely, when cells were activated by PHA, neither the PBMC proliferation (mainly, T-cells^+^) nor the LPI were affected by the BS extracts (Figures 3(a) and 3(c)).

It has been previously reported that the BS could produce opposing effects on immune responses in vivo or in vitro. Potentially low concentrations of BSs increase stimulated proliferation of lymphocytes whereas higher concentrations are even inhibitory [14].

Sharma et al. [38] reported that a mixture of various boswellic acids in the range of 1.95–125 *μ*g/mL inhibited mice splenocytes stimulated with lipopolysaccharides (LPS), PHA, alloantigen, and concanavalin A (ConA), in a concentration-dependent manner. Indeed, a significant inhibition of splenocytes to mitogens and alloantigens was observed starting from concentrations greater than 3.90 *μ*g/mL.

On the other hand, Gayathri et al. [15] observed that 30 *μ*g/mL of a crude methanolic BS extract is able to inhibit almost 80% of human lymphocyte proliferation. These data are in striking contrast with the observations of other authors [39] who tested the effect of 1 mg/mL of BS total alcoholic extract, gum, or volatile oil on human lymphocyte proliferation and observed no inhibition of cells stimulated with either PHA or Con A.

Besides the different lymphocyte proliferation assays applied, the BS concentrations used in the present study were 10 to 1000 times lower than those adopted in the cited articles and, at these doses, we obtained an effect on the lymphocyte proliferation only when cells were stimulated by PWM (B cells^+^).

However, in mice treated with orally administered boswellic acids, the secondary antibody titres were appreciably enhanced at the lowest tested doses (25 mg/kg body weight versus 100–200 mg/kg) [38].

The dose of BS extracts we adopted was probably low enough to induce an effect on B cells^+^ (activated by PWM), but too low to induce an inhibition of T lymphocyte proliferation, as reported by other authors [14, 39].

In the maintenance of T-cell balance a pivotal role is attributed to T-helper cells and regulatory T-cells [40, 41]. T-helper cells are defined as Th1-, Th2-, or Th17-cells and are characterized by differential expression of certain cytokines [42]. Th1-cells have the capacity to express the key cytokine interferon-*γ* (IFN-*γ*), whereas Th17-cells, a more recently described T-helper cell subset, evolutionally and functionally divergent from Th1 and Th2 cell subsets, are characterized by their ability to produce interleukin-17A (IL-17A) [43].

Regulatory T-cells (Tregs) suppress effector T-cells and, in humans, can be characterized by a CD4^+^CD25^high^FoxP3^+^ phenotype [41].

In our study, we observed that when PBMCs from healthy controls were not activated by PHA mitogen, the presence of BS extract G in the culture medium determined a significant increase of Tregs (Figure 4(a)). Furthermore, the increased number of Treg cells in BS G treated samples was accompanied by a higher number, although not significant, of Th17^+^ cells. Conversely, the BS extracts did not influence the number of Th1^+^ cells (INF*γ*^+^). When PBMCs were pulsed by PHA, no additional effect could be seen following the BS extract addition.

The recent evidence of a developmental plasticity between Treg and Th17 cells prompts the investigation of intermediate phenotypes that result from their reciprocal conversion according to the surrounding microenvironment [44]. The presented results show a possible role for BS extracts in such a fine balance between these two cell subsets.

Furthermore, it is important to note that, at least in mice, Th17 lymphocytes can also function as B-cell helpers [45], mediating B-cell differentiation and antibody class switch recombination. The results of our lymphocyte proliferation assay showed that the BS extracts exerted a significant stimulatory effect on B+ cell proliferation, possibly mediated by an enhanced number of Th17^+^ cells.

Many authors have demonstrated that* B. serrata* extracts turn out to be effective in the treatment of diseases such as inflammatory bowel disease and osteoarthritis in which inflammation and/or oxidative stress exert an important pathogenic role [2, 13, 30, 46].

However, BS extracts also exerted beneficial effects in some autoimmune diseases, such as rheumatoid arthritis [1], where chronic inflammation and an aberrant autoimmune response are hallmarks of the disease [47].

This* ex vivo* study provides evidence that* B. serrata* extracts, besides their reported capacity in dampening the inflammatory response together with counteracting the oxidative stress, were able to influence the regulatory and effector T-cell compartments.

In order to draw conclusions, it will be necessary to deepen the experiment on a wider case study. However, these preliminary results suggest that frankincense should be further investigated for its promising ability to interfere, possibly also through such regulatory mechanism, on immune dysregulation typical of various immune disorders, but attention should be paid to the quality of the commercial extracts which can show wide variations in their chemical composition.

## Figures and Tables

**Table 1:** AKBA quantification in *Boswellia serrata* extracts. Data are reported as mean ± SD (*n* = 3). Samples A–F = powder extracts, sample G = hydroenzymatic extract.

Sample	Concentration^§^	% in *B. serrata* extract^#^
A	38.30 ± 1.01	3.83 ± 0.10
B	17.18 ± 0.05	1.72 ± 0.005
C	3.08 ± 0.06	0.31 ± 0.01
D	24.35 ± 1.87	2.43 ± 0.19
E	nd^*∗*^	nd
F	21.07 ± 0.16	2.11 ± 0.02
G	0.29 ± 0.04	0.03 ± 0.004

^§^AKBA concentration is expressed as mg/g of powder extract, with the exception of sample G (mg/mL of hydroenzymatic extract); ^**#**^AKBA percentage is expressed as g/100 g of powder extract, with the exception of sample G (g/100 mL of hydroenzymatic extract); ^*∗*^nd = not detectable.

**Table 2:** In vitro radical scavenging activity and polyphenolic content of different *Boswellia* extracts.

*Boswellia serrata* extracts	Polyphenols mg GAE/g	DPPH		ABTS		FRAP
		TEAC^a^ *μ*mol TE/g	IC_50_^b^ *μ*g/ml	TEAC *μ*mol TE/g	IC_50_ *μ*g/ml	TEAC *μ*mol TE/g
A	7.68 ± 0.9	31.8 ± 0.7	340.2 ± 3.5	151.8 ± 10.6	79.26 ± 1.8	66.89 ± 3.5
B	1.43 ± 0.5	4.48 ± 0.08	2416 ± 12.5	37.54 ± 2.5	320.30 ± 4.5	ND
C	0.56 ± 0.2	3.83 ± 0.08	2823 ± 27.5	1.92 ± 0.1	6250 ± 17.5	ND
D	1.09 ± 0.4	6.29 ± 0.12	1720 ± 13.8	27.8 ± 1.9	431.62 ± 6.5	ND
E	1.09 ± 0.3	5.41 ± 0.11	1998 ± 17.5	18.7 ± 1.2	641.75 ± 9.5	ND
F	0.96 ± 0.3	5.20 ± 0.15	2080 ± 22.5	20.6 ± 21.4	581.94 ± 8.5	ND
G	0.11 ± 0.05	1.85 ± 0.03	5820 ± 32.5	1.76 ± 0.1	6800 ± 22.5	ND

*Positive control*						
Trolox			10.85 ± 0.2		3.01 ± 0.2	

^a^TEAC = Trolox equivalent (TE) antioxidant concentration. ^b^IC_50_ = The concentration of compound that affords a 50% reduction in the assay; GAE = gallic acid equivalent. ND = not detectable.
